# Neuropsychiatric Effects of Oseltamivir in an Older Adult With End-Stage Renal Disease: A Case Report

**DOI:** 10.7759/cureus.107825

**Published:** 2026-04-27

**Authors:** Kristie Y Hsu, Michi Yukawa

**Affiliations:** 1 Department of Medicine, VA San Diego Healthcare System, San Diego, USA; 2 Division of Geriatrics, Gerontology, and Palliative Care, Department of Medicine, University of California San Diego, San Diego, USA; 3 Division of Geriatrics, Department of Medicine, University of California San Francisco (UCSF) Medical Center, San Francisco, USA

**Keywords:** delirium, dementia, end-stage renal disease (esrd), nursing home, oseltamivir

## Abstract

Oseltamivir is a renally cleared antiviral medication that is highly effective and often prescribed for influenza prophylaxis in nursing home (NH) settings. We present the case of a 74-year-old man with a history of end-stage renal disease (ESRD) on hemodialysis (HD) and vascular dementia who received prophylactic oseltamivir during an influenza outbreak at his NH and subsequently developed acute neuropsychiatric symptoms. A comprehensive work-up for an acute reversible cause was not identified other than the introduction of the new medication, oseltamivir. The patient’s symptoms resolved after cessation of the medication. To our knowledge, we are the first to report a case of oseltamivir-induced delirium in an older adult receiving ESRD-dosed prophylactic oseltamivir. Our case highlights the need for providers to be vigilant regarding the potential neuropsychiatric side effects of oseltamivir among older adults receiving influenza prophylaxis. Older adults living with ESRD or dementia may be particularly vulnerable.

## Introduction

Oseltamivir is a renally cleared medication first approved by the United States (U.S.) Food and Drug Administration (FDA) in 1999. More recently, in 2022, it was prescribed 1.97 million times in the U.S. [1]. Oseltamivir is indicated for both the treatment and prevention of influenza and works by inhibiting neuraminidase activity, which thereby prevents viral replication. While oseltamivir is generally well-tolerated, in 2006 a warning was added to the FDA drug label after more than 2,000 reports of neuropsychiatric adverse events (NPAEs) - primarily among children and adolescents in Japan - raised concerns about potential adverse central nervous system effects [2,3].

In the U.S., the Centers for Disease Control and Prevention (CDC) recommends antiviral prophylaxis for all residents in nursing homes (NHs) experiencing an influenza outbreak [4]. Oseltamivir is commonly used in NHs due to these national recommendations and strong evidence demonstrating its high efficacy in preventing influenza [5].

Despite its widespread use, there are few published reports describing the neuropsychiatric effects of oseltamivir in older adults [6-8], particularly at the prophylactic dose used in NHs and in patients requiring end-stage renal disease (ESRD) dosing. In older adults, potential mechanisms of oseltamivir-induced delirium may include reduced renal clearance leading to higher central nervous system exposure to the active metabolite and age-related blood-brain barrier permeability [9,10]. To our knowledge, no previous report has documented neuropsychiatric effects from oseltamivir prophylaxis in an older adult with ESRD. This case adds to the literature a patient story that highlights the need to recognize the potential NPAEs of oseltamivir in vulnerable populations.

## Case presentation

A 74-year-old man with a history of vascular dementia and ESRD secondary to hypertension, on hemodialysis (HD) for 14 years, presented with acute neuropsychiatric symptoms in the NH after receiving prophylactic oseltamivir. He also had a past medical history of prior stroke with residual one-sided weakness and contractures, epilepsy, atrial fibrillation on anticoagulation, glaucoma, and pancreatic insufficiency.

**Figure 1:**
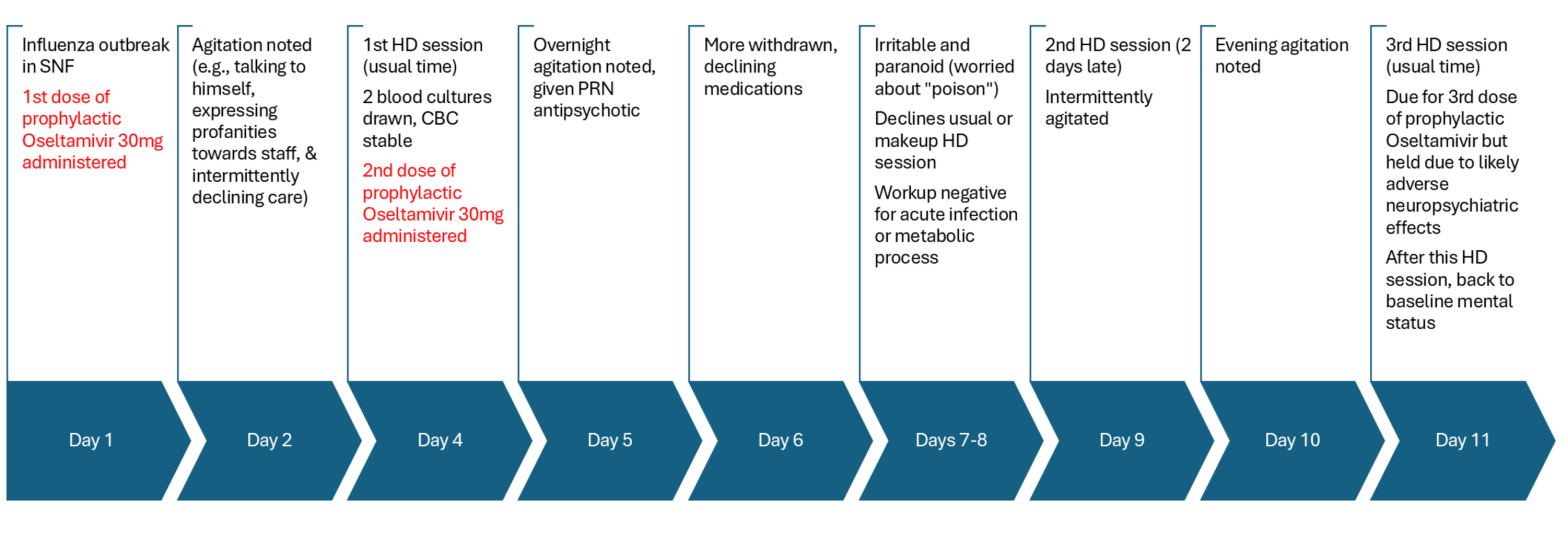
Summary of nursing home illness course Abbreviations: SNF, skilled nursing facility; CBC, complete blood count; HD, hemodialysis; PRN, as needed.

Figure 1 summarizes the NH course of illness. On day 1, the NH was placed on influenza outbreak status and required anti-viral prophylaxis for all residents. The patient was prescribed standard ESRD-dosing for prophylactic oseltamivir (30 mg on day 1, then 30 mg after every other HD session thereafter, for a total prophylaxis duration of 14 days). On day 2, the patient began to exhibit new paranoia and agitation, including talking to himself, expressing profanities towards staff, and intermittently declining care. His vital signs remained normal, with a pulse of 77, oxygen saturation of 98%, blood pressure of 103/63, and temperature of 97.3°F. On day 4, he went to his usual HD session and received his second dose of oseltamivir 30 mg. Over the following days, the patient exhibited fluctuating symptoms, including episodes of agitation (e.g., cursing at or throwing items at staff), as well as periods of withdrawal and paranoia (e.g., expressing concern about being poisoned), leading to intermittent refusal of care. Due to these mood symptoms, he repeatedly declined HD until his family persuaded him to attend an HD session two days later than scheduled.

**Figure 2:**
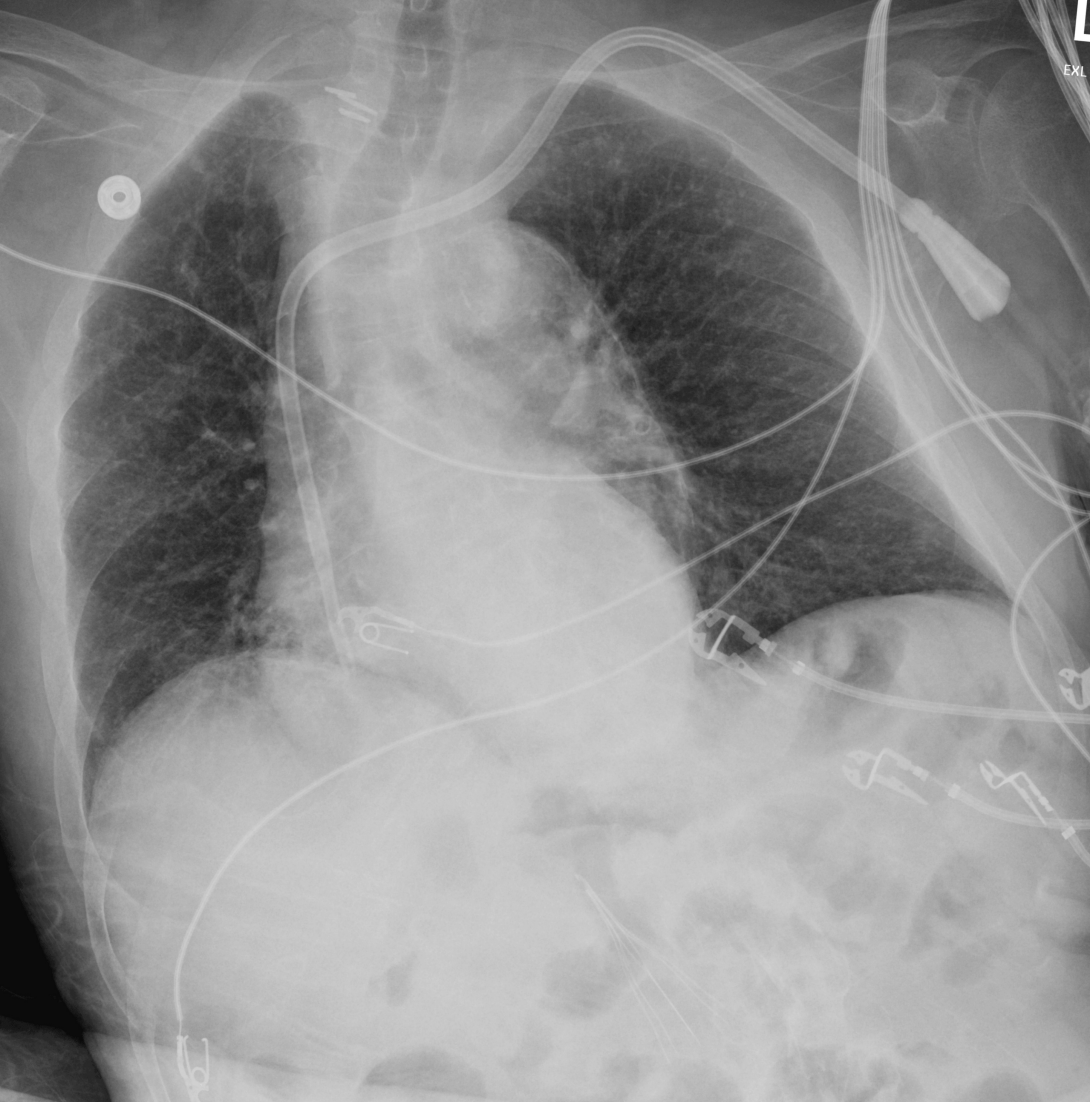
Chest X-ray

During this period of agitation, his vital signs remained normal. On physical exam, he had stable one-sided weakness and contractures from a prior stroke and no new focal neurological deficits to suggest a new cerebrovascular event. Aside from the neuropsychiatric alterations, he had no new symptoms concerning for infection. His lungs were clear, and his oxygen saturation ranged from 97% to 100% on room air. A subsequent chest X-ray did not show evidence of pneumonia (Figure 2). The NH pharmacist completed a detailed medication review, with oseltamivir being the only new prescription, and no additional drug-drug interactions were identified. He was anuric at baseline and denied any suprapubic discomfort. Blood cultures and complete blood counts were drawn twice (once on day 4 and once on day 8) and were unrevealing. A comprehensive metabolic panel drawn on day 8 was also unrevealing for an acute reversible cause of the neuropsychiatric disturbances (sodium 136 mmol/L, potassium 4.4 mmol/L, chloride 100 mmol/L, CO₂ 19 mmol/L, blood urea nitrogen (BUN) 94 mg/dL, creatinine 10.35 mg/dL, albumin 4.1 g/dL, phosphorus 8.2, alkaline phosphatase (ALP) 111 U/L, aspartate aminotransferase (AST) 7 U/L, alanine aminotransferase (ALT) 15 U/L, total bilirubin 0.3 mg/dL) and was reassuring against the need for emergent dialysis after delaying his normally scheduled HD session by two days.

Due to the absence of an identifiable acute infectious or metabolic cause of the patient’s neuropsychiatric symptoms, the decision was made to hold the third dose of oseltamivir that was originally scheduled for after his third HD session. He experienced complete resolution of his neuropsychiatric symptoms and returned to his baseline cognitive status after this third HD session. Oseltamivir was added to his list of allergies, and intranasal zanamivir (with careful monitoring) is considered an alternative agent should a future influenza outbreak occur.

## Discussion

We present a case of acute-onset neuropsychiatric symptoms in an older adult who received prophylactic oseltamivir during an NH influenza outbreak. This case highlights the importance of clinician vigilance regarding potential NPAEs associated with oseltamivir in vulnerable populations.

Oseltamivir is generally considered a well-tolerated medication, with the most commonly reported side effects being headache (up to 17%) and gastrointestinal symptoms, such as nausea (up to 16%) [11]. While NPAEs - such as delirium, hallucinations, and behavioral changes - have been previously described, these events are rare (<1%) and are reported predominantly in children [8,11,12]. A particularly high number of NPAEs have been documented among adolescents in Japan, where two teenagers tragically died by suicide while taking the antiviral. In contrast, reports of NPAEs among older adults are rare. To date, only three published reports have described NPAEs in older adults related to oseltamivir use - two involving treatment dosing for confirmed influenza and only one involving prophylactic dosing (Table 1) [6-8]. The mechanism underlying NPAEs remains incompletely understood but is thought to involve inhibition of nicotinic acetylcholine and monoamine oxidase-A receptors. Additional proposed mechanisms include modulation of NMDA and GABA receptors (similar to the action of ketamine) or stimulation of D2 dopaminergic receptors, which may contribute to altered neurochemical signaling and behavioral changes [9,10].

**Table 1:** Summary of case reports of oseltamivir-induced neuropsychiatric symptoms in older adults Abbreviations: ESRD, end-stage renal disease; HD, hemodialysis; MCI, mild cognitive impairment; CVA, cerebrovascular accident; CKD, chronic kidney disease.

Article	​Reason for Oseltamivir	Location	Age and Gender	Co-morbidities	​Symptoms and Resolution
Kohen (2007) [6]	Treatment	Nursing Home	83-year-old male	Vascular dementia	Admitted to inpatient psychiatric unit for agitation, aggression, and confusion. Symptom onset ~48 hours after starting medication. Symptoms resolved within 48 hours of medication cessation.​
Kader et al. (2024) [8]	Treatment	Hospital	74-year-old female	ESRD on HD, hypertension, COPD	Developed acute-onset hallucinations and delirium within 48 hours of starting medication. Symptoms resolved within a “few days” after cessation of medication.
Clifford et al. (2020) [7]	Prophylaxis	Nursing Home	74-year-old male	MCI, CVA, CKD3	Developed new psychiatric symptoms after receiving two doses of medication. Symptoms resolved after cessation of medication, but returned when the patient was “re-challenged” with medication. The symptoms again resolved after the second cessation of the medication.

Our case adds to the limited literature by describing the neuropsychiatric symptoms associated with oseltamivir prophylaxis in an older adult with multimorbidity. Like the case from Kader et al. [8], our patient had ESRD requiring dialysis, and, like the case from Kohen [6], he also had vascular dementia. In all reported cases, including ours, the neuropsychiatric symptoms resolved after discontinuation of oseltamivir.

It is important to note that the populations that may be more vulnerable to NPAEs (i.e., older adults living in a healthcare institution with comorbidities such as ESRD and cognitive impairment) are also those at greatest risk for morbidity and mortality from influenza. Given the growing demographic of older adults living in NHs or other group settings worldwide, it is important to understand the risks of the rare yet concerning NPAEs associated with oseltamivir. It is equally important to note that oseltamivir remains a highly effective agent for influenza prophylaxis, especially in these high-risk environments. Studies have shown that oseltamivir prophylaxis can reduce the risk of influenza infection by over 90% among older adult NH residents [5]. Both the CDC and the Infectious Diseases Society of America (IDSA) continue to recommend antiviral prophylaxis for all residents in long-term care facilities during an influenza outbreak [4,13]. Oseltamivir remains a life-saving intervention, and chemoprophylaxis in institutional settings is essential for reducing influenza-related morbidity and mortality.

Therefore, while clinicians should pay attention to potential NPAEs, our findings should not deter the use of oseltamivir when clinically indicated. Instead, they highlight the importance of individualized risk-benefit assessments and close monitoring for vulnerable patients. In our case, the decision to stop the antiviral was made because the adverse effects led to the patient refusing dialysis, which posed a significant health risk. However, in other cases, the neuropsychiatric symptoms may be mild, and the benefits of continued chemoprophylaxis could outweigh the risks. For our patient, should a future influenza outbreak occur, an alternative FDA-approved antiviral prophylactic agent, such as inhaled zanamivir, will be considered with careful monitoring, given his prior adverse reaction to oseltamivir.

Current prescribing information for oseltamivir from Lexicomp only warns of neuropsychiatric effects in children and adolescents aged 10-19 years and in males [11]. Our case, along with the limited literature available, suggests that additional risk factors - such as chemoprophylactic dosing [12], NH residence, cognitive impairment, and chronic kidney disease - may also increase susceptibility. Further research is needed to better define these potential risk factors and guide clinical decision-making in this vulnerable population.

## Conclusions

This case highlights the need for prescribers and frontline healthcare staff to be vigilant regarding the rare, yet potentially severe, neuropsychiatric side effects of oseltamivir in older adults receiving influenza prophylaxis, particularly among those with ESRD and cognitive impairment. Careful monitoring and strong interdisciplinary communication are essential for the prompt identification of neuropsychiatric changes that may be due to drug-induced delirium. This case further emphasizes the need for clinicians to guide individualized risk-benefit assessments, as the frail populations who may derive the most benefit from influenza prophylaxis are likely those most vulnerable to drug-related harm. Future studies are warranted to better characterize the predisposing risk factors for oseltamivir-induced delirium among older adults and whether alternative agents (e.g., zanamivir) may carry lower risks.
